# Association between platelet, white blood cell count, platelet to white blood cell ratio and sarcopenia in community-dwelling older adults: focus on Bushehr Elderly Health (BEH) program

**DOI:** 10.1186/s12877-022-02954-3

**Published:** 2022-04-08

**Authors:** Mohamad Gholizade, Akram Farhadi, Maryam Marzban, Mehdi Mahmudpour, Iraj Nabipour, Mohammadreza Kalantarhormozi, Gita Shafiee, Afshin Ostovar, Bagher Larijani, Amir Hossein Darabi, Eisa Safavi

**Affiliations:** 1 The Persian Gulf Tropical Medicine Research Center, The Persian Gulf Biomedical Sciences Research Institute, Bushehr University of Medical Sciences, Bushehr, Iran; 2 Department of Health Education and Promotion, Faculty of Health, Bushehr University of Medical Sciences, Bushehr, Iran; 3 Department of Biostatistics and Epidemiology, Faculty of Health and Nutrition, Bushehr University of Medical Sciences, Bushehr, Iran; 4 Department of Internal Medicine, School of Medicine, Bushehr University of Medical Sciences, Bushehr, Iran; 5 Chronic Diseases Research Center, Endocrinology and Metabolism Population Sciences Institute, Tehran University of Medical Sciences, Tehran, Iran; 6 Osteoporosis Research Center, Endocrinology and Metabolism Clinical Sciences Institute, Tehran University of Medical Sciences, Tehran, Iran; 7 Endocrinology and Metabolism Research Center, Endocrinology and Metabolism Clinical Sciences Institute, Tehran University of Medical Sciences, Tehran, Iran; 8 Department of Paraclinic, Bushehr University of Medical Sciences, Bushehr, Iran

**Keywords:** Sarcopenia, Platelet, White blood cell, Chronic inflammation, Older adults, Platelet to white blood cell ratio

## Abstract

**Background:**

Sarcopenia is a progressive age-related skeletal muscle disorder associated with harmful impacts on health. The present study aimed to investigate the relation between sarcopenia, platelet (PLT), white blood cell (WBC), and PLT to WBC ratio (PWR) due to the importance of early sarcopenia diagnosis.

**Methods:**

This cross-sectional study was conducted based on the second stage of the Bushehr Elderly Health (BEH) Program. Sarcopenia was defined based on the revised edition of the European Working Group on Sarcopenia in Older People (EWGSOP2) in accordance with the Iranian cut-off point. Univariate and adjusted multivariate logistic regression and linear regression were used to evaluate the associations.

**Results:**

The prevalence of sarcopenia among participants was 35.73%. PLT count and PWR were statistically higher in severe sarcopenic participants, while no differences were seen in WBC. In crude analysis, sarcopenia was not associated with quartiles of PLT, WBC, and PWR, while after adjusting for age, marital status, and sex, the association was seen in the fourth quartile of PLT and PWR [OR (95%CI) = 1.40 (1.08 to 1.81), *p*-value = 0.009 for PLT; OR (95%CI) =1.55 (1.20 to 2.00), *p*-value =0.001 for PWR]. This association remained significant in the fully adjusted model [OR (95%CI) =1.82 (1.20 to 2.78), *p*-value =0.005 for PLT; OR (95%CI) =1.57 (1.03 to 2.40), *p*-value =0.035 for PWR]. Among sarcopenia parameters, PLT count was more likely to be associated with handgrip strength and muscle mass. After stratifying the participants by gender, sarcopenia parameters were no longer statistically significant in men.

**Conclusion:**

This study showed that PLT and PWR were associated with sarcopenia after considering confounding factors, while this association was not seen in WBC. Moreover, results showed that gender had an important impact on sarcopenia parameters.

**Supplementary Information:**

The online version contains supplementary material available at 10.1186/s12877-022-02954-3.

## Introduction

Given the increase in the older adults’ population, the age-related diseases have proportionately raised [1]. On the other hand, aging has been shown to be associated with a decrease in muscle mass and muscle strength. It is estimated that each person, after age 30, approximately loses 0.1 to 0.5% muscle mass per year, which will escalate after age 65 [2, 3]. This age-related decline in muscle mass is defined as sarcopenia [4]. Sarcopenia is prevalent among older adults; however, its prevalence varies among older adults in various parts of the world [5]. Various mechanisms have been described for age-related muscle mass decline or sarcopenia, including insulin resistance, nutrition, age-related sex hormone, oxidative stress, neuromuscular dysfunction, endocrine abnormality, physical inactivity [6–8], but lately, studies have emphasized the key role of chronic inflammation in age-related muscle mass decrease [9]. It has been confirmed that aging is associated with a reduction in the regulation of pro-inflammatory cytokines [10]. This dysregulation is associated with decreased muscle mass and strength by interfering with muscle synthesis and catabolism [10, 11].

WBC, PLT, and platelet to white blood cell ratio count are broad and affordable disease indicators used in clinical settings. WBC is a standardized and stable marker that measures inflammation [12].

PLTs, a significant part of blood, have been shown that alongside the hemostasis function contribute to subclinical inflammation and oxidative stress [13]. Studies have shown that PLT activity increases in inflammatory diseases, and it has been confirmed that PLTs can indicate an inflammatory state [14]. Moreover, in vivo studies have demonstrated that age-related elevated TNF-α increases PLT activity, while anti-TNF-α administration declines PLT activity [15].

The results of previous studies for finding the association between sarcopenia and WBC [16–20], PLT [18, 20, 21], and the ratio of the type of WBC including neutrophil-to-lymphocyte ratio (NLR) [20, 22–25], lymphocyte-to-monocyte ratio (LMR) [25], and type of WBC to the PLT such as platelet-to-lymphocyte ratio (PLR) [22, 25–27], were controversial. Some of them did not find any significant result [25]; however, others found a significant association in the crude analysis, while they were not adjusted for confounding factors [23] or after considering other variables in the analysis were no longer associated with sarcopenia [19]. The others showed significant association in univariate and multivariate analysis [16, 18, 21, 26].

Among previous studies, The majority of them defined sarcopenia just based on muscle mass and did not consider muscle strength and physical ability [16, 18, 26, 27], which constitute indispensable parts of sarcopenia as well, while other studies which use both muscle mass and muscle strength had several limitations, including only assessing univariable association [23], perform on the population that their underlying disease affect outcomes and ignorance of obese sarcopenia [22], different methods for measuring muscle mass [22, 23].

Given the controversial results in previous studies and lack of sufficient information in eastern Mediterranean countries, and drawing on data from the BEH program, in this study, it was attempted to investigate whether PLT, WBC, and PWR are associated with sarcopenia. In addition, early diagnosis of sarcopenia with inexpensive markers like CBC seems to be essential for early detection, prevention, and treatment of sarcopenia.

## Methods

### Research design and participants

This cross-sectional study was conducted based on the second stage of the Bushehr elderly health (BEH) program. The methodology of the BEH program has previously been reported in detail [28, 29]. The BEH program is a prospective cohort study in Bushehr, south of Iran, targeting a population of 60 and over. Among 3297 who were selected through multistage stratified random sampling, 3000 were accepted to participate in the first phase of the study (participants rate was 91%). The first stage was conducted from March 2013 to October 2014, and the second phase, focusing on musculoskeletal and cognitive outcomes, started in 2015 with 2368 who were following the first stage (response rate was 81%).

### Measurement of laboratory parameters

Venous blood samples were collected from the participants following 8–12 h of fasting condition. Red blood cell count (RBC), hemoglobin (Hgb), WBC, PLT were assessed by an automated hematology analyzer, Medonic CA620 (Menarini Diagnostic Srl, Florence, Italy). Blood urea nitrogen (BUN), creatinine (Cr), uric acid, alkaline phosphatase (Alk-p), fasting plasma glucose (FPG), and lipid profile were assessed by an auto-analyzer using commercial kits (ParsAzmun, Karaj, Iran). Hemoglobin A1c (HbA1c) was measured by the CERA-STAT system (CERAGEMMEDISYS, chungcheongnam-do, Korea).

### Measures and definition of sarcopenia

Sarcopenia was defined based on the current revised edition of the European Working Group on sarcopenia in Older People (EWGSOP2), issued recently and defined as having low muscle mass and low muscle strength; it is also characterized as severe if the previous criteria were extant with poor physical performance. Dual x-ray absorptiometry (DXA, Discovery WI, Hologic, Bedford, Virginia, USA) was used to measure fat mass and muscle mass with minimal radiation exposure. Appendicular skeletal muscle mass (ASM) for each participant was calculated as the sum of upper and lower limb muscle mass. The skeletal muscle mass index (SMI) was defined as ASM/height^2^ (kg/m^2^). According to previous studies, the cut-off point for low muscle mass was defined as SMI < 7.0 kg/m^2^ for men and < 5.4 kg/m^2^ for women in the Iranian population [30]. Muscle strength was assessed based on handgrip strength and chair stand measures. Handgrip strength was measured three times for each hand using a digital dynamometer. The handgrip strength threshold was 26 kg for men and 18 kg for women [30]. In this study, the chair stand test was used to assess the lower extremity muscle strength [31]. For the measuring chair stand test, participants were asked to keep their arms folded across their chest; then, if participants could perform the first test, they were asked to stand up and sit down five times without using arms. Time was recorded for each participant from the initial sitting to the final standing position, and the cut-off point was defined as chair stand test time > 15 s. Physical performance was evaluated by short physical performance battery (SPPB) and usual gait speed. SPPB is a group of tests evaluating physical performance by combining the result of the chair stand, gait speed, and balance tests described elsewhere [29]. For measuring the usual gait speed, participants were asked to walk for 4.57 m at a normal pace twice; then, the fastest record was used. Poor physical performance was defined as SPPB ≤8 point score or gait speed ≤0.8 m/s [31].

### Other variables

Metabolic syndrome (MetS) was defined according to the revised edition of national cholesterol education program adult treatment panel III (NCEP-ATP III) [32], and cognitive function was assessed using the mini-mental state examination (MMSE), mini-cog, and categorical verbal fluency test (CFT), which have been described in the previous study [33]. For CFT, we used a cut-off point of 14 for those who had completed elementary school and 12 for those who had completed less than 5 years of education. For the MMSE,20 and 24 were considered cut-off points for those with an education level lower and higher than the primary school, respectively. For mini-cog, the test had two parts, recalling three-word, registrations, and recalling and drawing the clock test; also, the test was considered impaired if any participant could not recall all three words or draw the specific time in a clock. If the CFT, MMSE, or mini-cog scores were low, the participants were diagnosed with cognitive impairment. The chronic diseases included liver disease, lung disease, cardiovascular disease, thyroid diseases, osteoarthritis (OA), rheumatoid arthritis (RA), which were defined as self-reported or medication use. Chronic renal failure was defined as glomerular filtration rate (GFR) below 60; hypertension (HTN) as medication use, systolic blood pressure ≥ 140 mmHg, or diastolic blood pressure ≥ 90 mmHg), and diabetes mellitus (DM), as HbA1C ≥ 6.5, FPG ≥ 126 mg/dl or taking anti-diabetic medication). Use of Anti-inflammatory medication was defined as the implementation of non-steroidal anti-inflammatory drugs (NSAID), azathioprine, mesalazine, sulfasalazine, methotrexate, mycophenolate mofetil, corticosteroids, colchicine, and tacrolimus. Use of anti-PLT medication was defined as the use of aspirin (ASA), clopidogrel, and dipyridamole. The use of anti-hyperlipidemic medication comprised the implementation of statins (atorvastatin, lovastatin, and simvastatin) or fibrates (gemfibrozil and fenofibrate). The use of HTN medication was characterized by the implementation of angiotensin-converting enzyme inhibitors (ACEIs), angiotensin receptor blockers (ARBs), alpha-blocker medications, beta-blockers, calcium channel blockers (CCBs), diuretic medications, and nitrates medications. Other covariates included age (years), gender (male/female), marital status (single, married, divorced, and widow), and smoking, which included *no history* of smoking, *smoking regularly* if the participant had a history of smoking at least one cigarette per day in a week, and the *lower rate* known as smoking occasionally. Still, as other covariates, body mass index (BMI) was calculated by dividing weight (kg) to height squared (m^2^); waist to hip circumference ratio (WHR), which was calculated by dividing waist circumference (WC) to hip circumference (HC), and disability was assessed by instrumental activities of daily living (IADL) using Lawton scale questionnaires, translated into Persian [34]. IADL test consisted of eight items (use of telephone, shopping, meal preparation, housekeeping and laundry, mode of transportation, medication management, and money management) in daily living activities. The maximum score was eight and considered independent, while the score < 8 was interpreted as a value of dependency.

### Statistical analysis

The normality of all variables was assessed by the Kolmogorov–Smirnov test. PLT, WBC and PWR were divided into four quartiles as fellows: Q1 ≤ 220, 220 < Q2 < 259, 259 ≤ Q3 ≤ 300, and Q4 >  300 (10^3^/ *μl*) for PLT; Q1 ≤ 6.1, 6.1 < Q2 < 7.3, 7.3 ≤ Q3 ≤ 8.4, and Q4 >  8.4 (10^3^/ *μl*) for WBC, and Q1 ≤ 29.46, 29.46 < Q2 < 36, 36 ≤ Q3 ≤ 43.28, and Q4 >  43.28 for PWR. Categorical variables were presented by the frequency and percentage, and the mean and standard deviation (SD) were used for continuous variables. Differences in quartiles were evaluated by running one-way analysis of variance (ANOVA) and chi-square (X^2^) for continuous variables and categorical variables, respectively. Multivariable linear and logistic regression analyses were used to evaluate the association between PLT, sarcopenia, and sarcopenia parameters. To evaluate the association between PLT and sarcopenia parameters, we stratified participants based on gender, which helped clarify the effect of gender on sarcopenia parameters. In this study, missing values were not significant and were negligible. Covariates that had a significant clinical and pathophysiological association with desired outcomes were selected standardized base method and combination of previous studies finding and analyses, and epidemiologist and geriatric specialist [35]. The priority of confounder variables selection was based on their effect and association univariable analysis and previous studies. Covariates were adjusted as: **model 1** = age, marital, and gender; **model 2** = model 1 + smoking, metabolic syndrome, and the number of chronic diseases; **model 3** = model2 + Anti-inflammatory medications, anti-PLT medications, anti-diabetic medications, anti-hyperlipidemic medications, HTN medications, IADL, and BMI; **model 4** = model 3 + laboratory parameters (HGB, TG, and creatinine). Stata MP (version 15) was used, and a two-sided *p*-value of < 0.05 was taken as statistically significant for all analyses. *P*-values for trends were obtained from adjusted models by assigning quartiles as continuous variables. We used the STATA software by modifying the two-way lfitci command to make fractional polynomial plots by classifying sex for age, which is predicted by a linear prediction for PLT, and PWR. Moreover, for other figures, we used Prism version 8.00 (GraphPad Software, La Jolla California, USA), DAGitty v3.0.

## Results

**Fig. 1:**
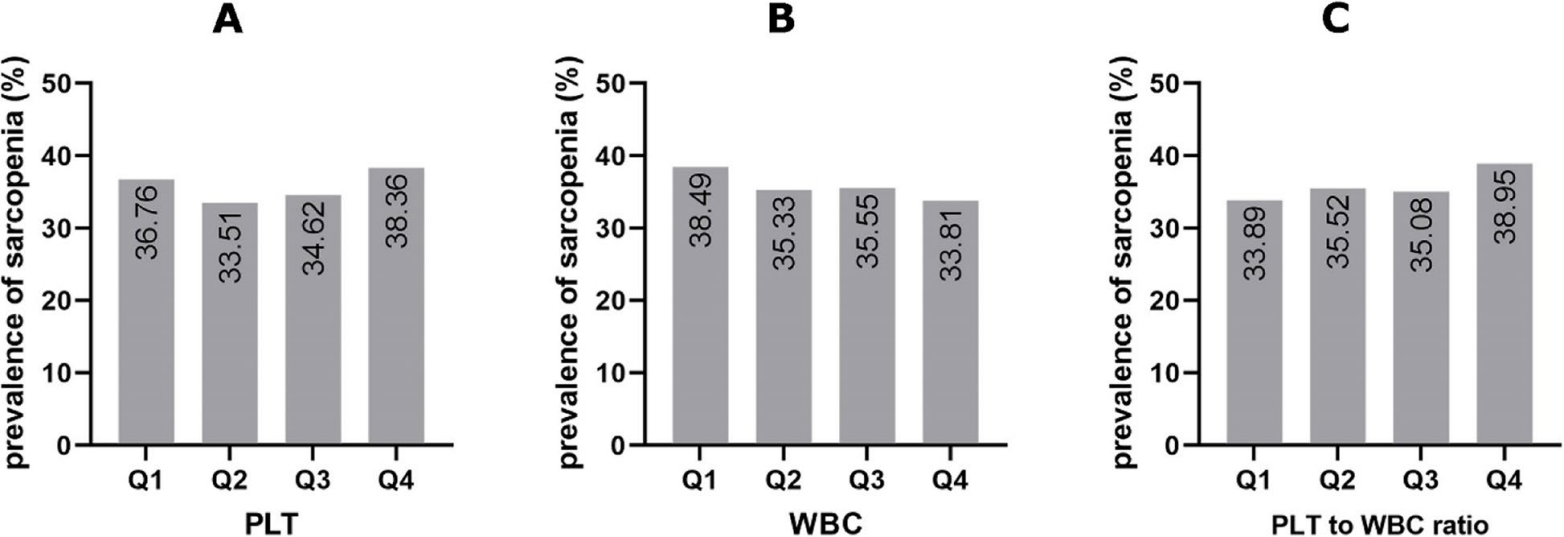
The prevalence of sarcopenia according to PLT, WBC, and PLT to WBC ratio quartiles. Prevalence of sarcopenia was presented in each quartile. **A** Quartiles for PLT were defined as fellow: Q1 ≤ 220, 220 < Q2 < 259, 259 ≤ Q3 ≤ 300, and Q4 > 300 (10^3^/ *μl*); **B** For WBC, quartiles were defined as Q1 ≤ 6.1, 6.1 < Q2 < 7.3, 7.3 ≤ Q3 ≤ 8.4, and Q4 > 8.4 (10^3^/ *μl*); for PLT to WBC ratio (PWR), quartiles were defined as Q1 ≤ 29.46, 29.46 < Q2 < 36, 36 ≤ Q3 ≤ 43.28, and Q4 > 43.28

Of 2368 who were included in this study, 1223 participants (51.65%) were female. The mean age of the participants was 69.34 ± 6.33, and the prevalence of sarcopenia among participants was 35.73%. According to Table 1, there are significant differences in PLT count and PWR between sarcopenic and nonsarcopenic participants, while no statistical differences are seen in WBC. Participants with severe sarcopenia have a higher prevalence of metabolic syndrome, cognitive disorder, and they are more likely to use anti-inflammatory, anti-hyperlipidemic, and anti-HTN medication than participants with mild sarcopenia. The prevalence of sarcopenia is presented according to PLT, WBC, and PLT to WBC ratio quartiles in Fig. 1.

**Table 1:** Characteristics of the study participants based on the severity of sarcopenia in the BEH program

	Parameters		Total (***n*** = 2368)			Severe sarcopenia (***n*** = 454)	*p*-value ^a^
				No sarcopenia (***n*** = 1522)	Mild sarcopenia (***n*** = 392)		
**Demographic & Clinical**	Age (years)		69.30 ± 6.33	67.99 ± 5.48	69.58 ± 6.05	73.44 ± 7.35	< 0.0001
	Sex (Female), n (%)		1223 (51.65)	836 (54.93)	132 (33.67)	255 (56.17)	< 0.001
	Marital status, n (%)	Single	19 (0.80)	10 (0.66)	2 (0.51)	7 (1.54)	< 0.001
		Married	1824 (77.03)	1189 (78.12)	331 (84.44)	304 (66.96)	
		Divorce	20 (0.84)	10 (0.66)	6 (1.53)	4 (0.88)	
		Widow	505 (21.33)	313 (20.57)	53 (13.52)	139 (30.62)	
	Smoking	None	1523 (83.41)	994 (83.95)	233 (76.90)	296 (87.32)	0.003
		Yes, occasionally	30 (1.64)	15 (1.27)	10 (3.30)	5 (1.47)	
		Yes, regularly	273 (14.95)	175 (14.78)	60 (19.80)	38 (11.21)	
	Cognitive disorder, n (%)		1397 (59.45)	860 (57.03)	212 (54.36)	325 (71.90)	< 0.001
	Metabolic syndrome, n (%)		1179 (49.81)	863 (56.70)	127 (**32.40**)	189 (41.72)	< 0.001
	Chronic disease	None	244 (10.30)	152 (9.99)	55 (14.03)	37 (8.15)	0.066
		One	317 (13.39)	199 (13.07)	53 (13.52)	65 (14.32)	
		Two or more	1807 (76.31)	1171 (76.94)	284 (72.45)	352 (77.53)	
	SBP (mm Hg)		139.57 ± 19.32	140.08 ± 18.83	136.44 ± 20.16	140.57 ± 19.98	0.0019
	DBP (mm Hg)		81.49 ± 8.64	82.10 ± 8.40	80.48 ± 8.74	80.29 ± 9.15	< 0.0001
	BMI (kg/m^2^)		27.33 ± 4.63	29.05 ± 4.44	24.19 ± 3.07	24.28 ± 3.16	< 0.0001
	Waist circumference (cm)		98.39 ± 11.67	101.81 ± 10.98	92.33 ± 10.13	92.16 ± 10.42	< 0.0001
	WHR		.89 ± .12	.90 ± .15	.89 ± .06	.88 ± .07	0.1193
	IADL (dependent), n (%)		1201 (56.31)	751 (53.84)	164 46.99)	286 (73.52)	< 0.001
**Sarcopenia parameters**	ASM (kg)		15.89 ± 3.63	16.77 ± 3.52	15.24 ± 3.13	13.51 ± 3.15	< 0.0001
	SMI (kg/m^2^)		6.23 ± .98	6.58 ± .90	5.78 ± .77	5.45 ± .76	< 0.0001
	Total body fat mass (%)		37.57 ± 8.11	38.71 ± 8.11	34.00 ± 7.58	36.83 ± 7.57	< 0.0001
	Gait speed (m/s)		.84 ± .30	.87 ± .31	1.05 ± .17	.59 ± .17	< 0.0001
	SPPB		9.38 ± 1.73	9.62 ± 1.72	9.61 ± 1.04	8.22 ± 1.86	< 0.0001
	Mean hand grip (kg)		22.22 ± 9.20	23.83 ± 9.64	22.17 ± 7.61	16.88 ± 6.59	< 0.0001
**Medications**	Anti-inflammatory medication, n (%)		1171 (60.27)	771 (60.71)	157 (53.22)	243 (64.29)	0.012
	AntiPLT medication, n (%)		995 (51.21)	661 (52.05)	137 (46.44)	197 (52.12)	0.205
	Antihyperlipidemia medication, n (%)		807 (41.53)	563 (44.33)	111 (37.63)	133 (35.19)	0.002
	Anti-HTN medication, n (%)		1186 (61.04)	794 (62.52)	153 (51.86)	239 (63.23)	0.002
	DM medication, n (%)		591 (30.42)	390 (30.71)	83 (28.14)	118 (31.22)	0.641
**Biochemical parameters**	Total cholesterol		182.13 ± 44.20	182.54 ± 44.14	181.38 ± 44.38	181.39 ± 44.33	0.8301
	HDL-cholesterol (mg/dl)		45.96 ± 11.20	45.67 ± 10.95	45.90 ± 10.99	46.99 ± 12.16	0.0873
	LDL- cholesterol		109.40 ± 37.75	109.07 ± 38.39	110.28 ± 36.88	109.74 ± 36.38	0.8340
	TG		135.69 ± 70.27	140.45 ± 69.57	127.92 ± 63.16	126.45 ± 76.77	0.0001
	Hgb		14.50 ± 1.73	14.56 ± 1.72	14.57 ± 1.65	14.21 ± 1.84	0.0004
	RBC (10^6)		5.01 ± .63	5.03 ± .62	5.03 ± .62	4.91 ± .69	0.0017
	PLT (10^3)		262.52 ± 66.02	262.10 ± 65.23	256.61 ± 67.75	269.02 ± 66.75	0.0224
	WBC (10^3)		7.36 ± 2.19	7.43 ± 2.36	7.16 ± 1.82	7.30 ± 1.84	0.0789
	PLT to WBC ratio		37.28 ± 11.34	36.94 ± 11.16	37.22 ± 11.18	38.48 ± 11.99	0.0402
	BUN		14.98 ± 5.70	14.59 ± 5.42	15.26 ± 5.31	16.07 ± 6.73	< 0.0001
	Creatinine		1.10 ± .36	1.08 ± .35	1.12 ± .31	1.13 ± .44	0.0292
	Uric acid		5.17 ± 1.30	5.21 ± 1.28	5.14 ± 1.26	5.08 ± 1.39	0.1882
	Alk-P		220.32 ± 75.80	222.23 ± 77.74	212.07 ± 74.28	221.05 ± 69.98	0.0593
	HbA1c		5.67 ± 1.56	5.69 ± 1.54	5.57 ± 1.47	5.66 ± 1.67	0.3843

*BEH* Bushehr elderly health, *SBP* Systolic blood pressure, *DBP* diastolic blood pressure, *BMI* Body mass index, *WHR* waist to hip ratio, *IADL* Instrumental activities of daily living, *ASM* Appendicular skeletal muscle mass, *SMI* skeletal muscle mass index, *SPPB* Short Physical Performance Battery, *HTN* Hypertension, *HDL* high-density lipoproteins, *LDL* low-density lipoproteins, *TG* triglycerides, *Hgb* Hemoglobin, *RBC* Red blood cells, *PLT* platelet, *WBC* White blood cells, *PWR* PLT to WBC ratio, *BUN* blood urea nitrogen, *ALK-P* Alkaline phosphatase, *HbA1c* hemoglobin A1c

^a^ *P*-values for continuous variables and categorical variables were assessed using ANOVA and Chi-square, respectively

Table 2 demonstrates multivariate logistic regression analysis between sarcopenia, PLT, WBC, and PWR. Considering the lowest quartile (Q1) as a reference, in unadjusted analysis, sarcopenia is not associated with PLT, WBC, and PWR. Although WBC is not associated with sarcopenia in any analysis models, the association between PLT, PWR, and sarcopenia appears after adjusting covariates. According to the analysis, the association between sarcopenia and the fourth quartile is seen after adjusting age, gender, marital status (model 1) [OR (95%CI) = 1.40 (1.08 to 1.81), *p*-value =0.009 for PLT; OR (95%CI) =1.55 (1.20 to 2.00), p-value =0.001 for PWR]. This association between PLT, PWR, and sarcopenia in the fourth quartile remained significant as other covariates were considered in the analysis [in the fully-adjusted model (model 4): OR (95%CI) =1.82 (1.20 to 2.78), p-value =0.005 for PLT; OR (95%CI) =1.57 (1.03 to 2.40), *p*-value = 0.035 for PWR].

**Table 2:** Associations between PLT, WBC, PWR, and sarcopenia parameters in the BEH program

Independent Variables	Quartiles									
PLT	**Analytic Model**	**Q1** ***≤*** **220**		**220** ***<*** **Q2 < 259**		**259*≤*Q3** ***≤*** **300**		**Q4** ***>*** **300**		***P*-value for trend**
		OR (95% CI)	*P*-value	OR (95% CI)	*P*-value	OR (95% CI)	*P*-value	OR (95% CI)	*P*-value	
	Unadjusted	1 (reference)	–	.86(.68 to 1.10)	0.242	.91 (.72 to 1.14)	0.433	1.07(.84to 1.34)	0.564	0.546
	Model 1	1(reference)	–	1.00 (.77 to 1.28)	0.987	1.14 (.88 to 1.46)	0.306	1.40 (1.08 to 1.81)	0.009	0.007
	Model 2	1(reference)	–	1.13 (.84 to 1.52)	0.390	1.24 (.92 to 1.66)	0.143	1.62 (1.20 to 2.18)	0.002	0.002
	Model 3	1(reference)	–	1.24 (.82 to 1.86)	0.300	1.46 (.98 to 2.18)	0.062	1.87 (1.23 to 2.84)	0.003	0.003
	Model 4	1(reference)	–	1.25 (.83 to 1.88)	0.275	1.45 (.97 to 2.17)	0.066	1.82 (1.20 to 2.78)	0.005	0.004
WBC	**Analytic Model**	**Q1** ***≤*** **6.1**		**6.1** ***<*** **Q2 < 7.3**		**7.3*≤*Q3** ***≤*** **8.4**		**Q4** ***>*** **8.4**		***P*-value for trend**
		OR (95% CI)	*P*-value	OR (95% CI)	*P*-value	OR (95% CI)	*P*-value	OR (95% CI)	*P*-value	
	Unadjusted	1 (reference)	–	.87 (.69 to 1.09)	0.248	.88 (.69 to 1.11)	0.295	.81 (.64 to 1.03)	0.093	0.112
	Model 1	1(reference)	–	.87 (.68 to 1.11)	0.281	.91(.71 to 1.16)	0.460	.82 (.64 to 1.05)	0.123	0.169
	Model 2	1(reference)	–	.86 (.65 to 1.14)	0.317	.94 (.71 to 1.25)	0.712	.95 (.71 to 1.27)	0.759	0.883
	Model 3	1(reference)	–	.85 (.61 to 1.19)	0.373	.82 (.59 to 1.15)	0.264	.93 (.66 to 1.29)	0.672	0.598
	Model 4	1(reference)	–	.86 (.59 to 1.27)	0.469	1.16 (.78 to 1.73)	0.444	1.13 (.76 to 1.67)	0.541	0.335
PWR	**Analytic Model**	**Q1** ***≤*** **29.46**		**29.46** ***<*** **Q2 < 36**		**36*≤*Q3** ***≤*** **43.28**		**Q4** ***>*** **43.28**		***P*-value for trend**
		OR (95% CI)	*P*-value	OR (95% CI)	*P*-value	OR (95% CI)	*P*-value	OR (95% CI)	*P*-value	
	Unadjusted	1 (reference)	–	1.07 (.84 to 1.36)	0.555	1.05 (.82 to 1.33)	0.666	1.24 (.98 to 1.57)	0.070	0.094
	Model 1	1(reference)	–	1.12 (.87 to 1.44)	0.361	1.19 (.92 to 1.538)	0.176	1.55 (1.20 to 2.00)	0.001	0.001
	Model 2	1(reference)	–	1.15 (.85 to 1.54)	0.345	1.31 (.97 to 1.76)	0.073	1.62 (1.20 to 2.18)	0.001	0.001
	Model 3	1(reference)	–	.99 (.66 to 1.48)	0.974	1.31 (.88 to 1.96)	0.178	1.62 (1.07 to 2.46)	0.022	0.009
	Model 4	1(reference)	–	.98 (.65 to 1.46)	0.922	1.31 (.88 to 1.96)	0.180	1.57 (1.03 to 2.40)	0.035	0.015

Multivariate logistic regression was used for analysis

Model 1 adjusted for age, marital, and sex

Model 2 adjusted for Model 1+ smoking, metabolic syndrome, and the number of chronic diseases^**a**^

Model 3 adjusted for Model 2 + anti-inflammatory medications, anti- platelet medications, anti-diabetic medications, anti-hyperlipidemic medications, HTN medications, IADL, and BMI

Model 4 adjusted for Model 3 + Hgb, TG, and creatinine

*BEH* Bushehr elderly health, *PLT* platelet, *WBC* White blood cells, *PWR* PLT to WBC ratio, *HTN* Hypertension, *IADL* Instrumental activities of daily living, *WHR* waist to hip ratio, *BMI* Body mass index, *Hgb* Hemoglobin, *HbA1c* hemoglobin A1c, *HDL* high-density lipoproteins, *ALK-P* Alkaline phosphatase, *TG* triglycerides

^**a**^Chronic diseases included: liver diseases, lung diseases, cardiovascular disease, Hypertension,, diabetes mellitus, thyroid diseases, osteoarthritis, and rheumatoid arthritis

Multivariate linear regression between PLT and sarcopenia parameters is illustrated in Table 3. Results show that after fully adjusted analysis (model 4), only the association between ASM and handgrip remains statistically significant, which means that PLTs are more likely to affect handgrip strength (β = −.0145) and ASM (β = −.0086) compared to gait speed (β = −.0001) and SPPB) β = −.0008). However, after stratified analysis based on gender, none of the sarcopenia parameters are statistically associated with PLTs in men (all *p*-values > 0.05), while in women, handgrip remains statistically significant even after adjusting for age, marital, smoking, metabolic syndrome, and chronic diseases (model 2), and ASM remains statistically significant in the fully adjusted model (model 4).

**Table 3:** The relationship between PLT and sarcopenia parameters in the Bushehr Health (BEH) Program

Outcome variable	Analytic Model	All		Male		Female	
		β (95% CI)	*P*-value	β (95% CI)	*P*-value	β (95% CI)	*P*-value
^a^							
ASM	Unadjusted	−0.0135 (−0.0157 to −.0114)	< 0.001	−0.0006(−0.0006 to 0.0019)	0.649	−0.0034 (−0.0053 to −0.0015)	< 0.001
	Model 1	−0.0118(− 0.0138 to − 0.0098)	< 0.001	− 0.0015(− 0.0039 to 0.0008)	0.206	−0.0034 (− 0.0052 to − 0.0015)	< 0.001
	Model 2	−.0113 (−.0135 to −.0090)	< 0.001	−.0024 (−.0050 to .0001)	0.069	−.0044 (−.0065 to −.0023)	< 0.001
	Model 3	−.0118(−.0141 to −.0094)	< 0.001	−.0025 (−.0051 to .0000)	0.054	−.0033 (−.0051 to-.0015)	< 0.001
	Model 4	−.0086 (−.0108 to −.0064)	< 0.001	−.0021 (−.0047 to .0003)	0.097	−.0033 (−.0051 to −.0015)	< 0.001
Handgrip	Unadjusted	−0.0300 (− 0.0354 to − 0.0245)	< 0.001	0.0002 (− 0.0072 to 0.0077)	0.947	−0.0047 (− 0.0091 to − 0.0003)	0.033
	Model 1	− 0.0262 (−.0311 to − 0.0212)	< 0.001	−.0030 (−.0096 to .0036)	0.370	−.0050(−.0089 to −.0010)	0.013
	Model 2	−.0229 (−.0284 to −.0174)	< 0.001	−.0044 (−.0118 to .0029)	0.241	−.0053 (−.0099 to −.0007)	0.023
	Model 3	−.0219 (−.0279 to −.0159)	< 0.001	−.0055 (−.0146 to .0035)	0.231	−.0040 (−.0090 to .0010)	0.118
	Model 4	−.0145(−.0204 to −.0087)	< 0.001	−.0042 (−.0133 to .0048)	0.359	−.0032 (−.0083 to .0018)	0.215
gait speed	Unadjusted	−.0004 (−.0006 to −.0003)	< 0.001	−.0000 (−.0002 to .0002)	0.901	−.0000 (−.0003 to .0001)	0.533
	Model 1	−.0004 (−.0006 to −.0002)	< 0.001	−.0001 (−.0003 to .0001)	0.332	−.0000 (−.0002 to .0001)	0.549
	Model 2	−.0003 (−.0005 to −.0001)	0.001	−.0000 (−.0003 to .0002)	0.905	−.0000 (−.0003 to.0001)	0.616
	Model 3	−.0002 (−.0004 to −.0000	0.043	−.0001 (−.0004 to .0002)	0.551	.0001 (−.0001 to .0003)	0.471
	Model 4	−.0001 (−.0003 to .0000)	0.188	−.0000 (−.0004 to .0002)	0.628	.0000 (−.0001 to .0003)	0.513
SPPB	Unadjusted	−.0024 (−.0035 to −.0013)	< 0.001	.0001 (−.0013 to.0016)	0.858	−.0010 (−.0027 to .0006	0.209
	Model 1	−.0022 (−.0032 to −.0012)	< 0.001	−.0002 (−.0016 to .0011)	0.698	−.0012 (−.0028 to .0003)	0.115
	Model 2	−.0020 (−.0032 to −.0008)	0.001	−.0000 (−.0016 to .0015)	0.915	−.0017(−.0036 to .0001)	0.068
	Model 3	−.0011 (−.0025 to .0002)	0.119	.0006 (−.0013 to .0025)	0.534	−.0006 (−.0027 to.0013)	0.522
	Model 4	−.0008 (−.0022 to .0006)	0.272	.0007 (−.0011 to .0027)	0.430	−.0008 (−.0029 to .0012)	0.447

Multivariate linear regression was used for analysis

Model 1 adjusted for age, marital

Model 2 adjusted for Model 1+ smoking, metabolic syndrome, and the number of chronic diseases^**b**^

Model 3 adjusted for Model 2 + anti-inflammatory medications, anti-PLT medications, anti-diabetic medications, anti-hyperlipidemic medications, HTN medications, IADL, and BMI

Model 4 adjusted for Model 3 + Hgb, TG, and Creatinine

*BEH* Bushehr elderly health, *ASM* Appendicular skeletal muscle mass, *SPPB* Short Physical Performance Battery; *HTN* Hypertension, *IADL* Instrumental activities of daily living, *WHR* waist to hip ratio, *BMI* Body mass index, *Hgb* Hemoglobin, *WBC* White blood cells, *HbA1c* hemoglobin A1c, *HDL* high-density lipoproteins, *ALK-P* Alkaline phosphatase, *TG* triglycerides

^a^ PLT concentration was used as an independent variable

^**b**^Chronic diseases included: liver diseases, lung diseases, cardiovascular disease, Hypertension, diabetes mellitus, thyroid diseases, osteoarthritis, and rheumatoid arthritis

**Fig. 2:**
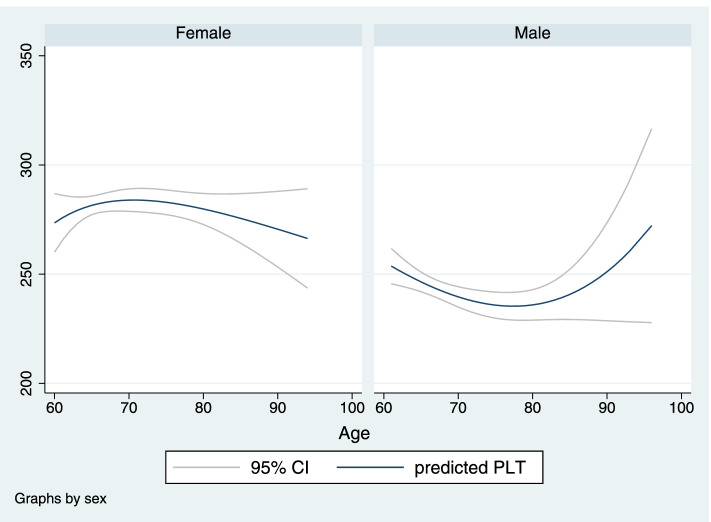
Fractional polynomial plot and 95% CI association between PLT and age

**Fig. 3:**
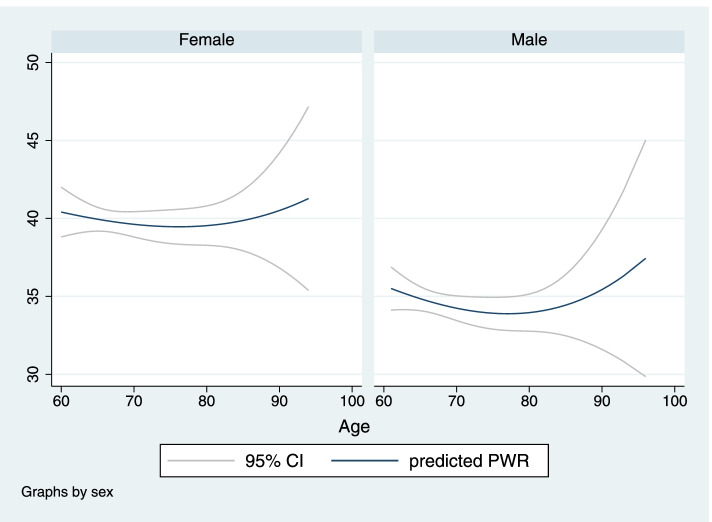
Fractional polynomial plot and 95% CI association between PWR and age

Figures 2 and 3 show the association between PLT, PWR, and age, based on gender differences by fractional polynomial plots. As figures illustrate, by increasing age, PWR escalates; however, PLT slightly declines in women over 90 years old.

## Discussion

This study showed that sarcopenia association with PLT count and PWR appeared in the primary adjustment, which remained significant even after controlling for potential confounders. This association was not seen between WBC and sarcopenia. Moreover, among sarcopenia measures, a prominent effect of PLTs was only seen in ASM and handgrip. However, considering gender separately, the association between PLT and sarcopenia parameters remained statistically significant only in women.

**Fig. 4:**
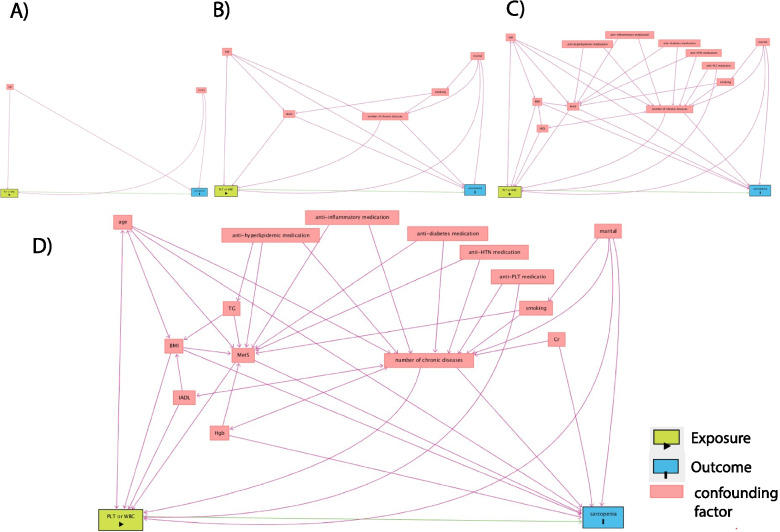
Directed acyclic graphs (DAGs) for assessing the effect of confounding variables. This graph demonstrates the effects of confounding factors on the association between sarcopenia and platelet (PLT) or white blood cell (WBC); **A** Model 1: Age + martial; **B** Model 2: model 1 + metabolic syndrome (MetS) + number of chronic diseases + smoking; **C** Model 3: model 2+ anti-plT+ ani-htn + anti-hypelimemic+ anti- inflammatory medication + *instrumental Activities of Daily Living* (*IADLs*) + BMI; D) Model 4: model 3 + hemoglobin(Hgb) + triglyceride (TG) + creatinine (Cr)

According to previous studies, age and marital status were used as important demographic factors in model 1 [16, 21, 26, 36, 37]. We also use metabolic syndrome, the number of chronic diseases, smoking in the second model as confounder variables [16, 18, 20, 26, 38, 39]. In model 3, we use medication related to chronic diseases that showed that controlled chronic diseases contribute to sarcopenia and directly affect sarcopenia parameters and platelet function [40–44]. In model 3, we also use BMI and IADL as confounding factors due to the previous finding, which is showed that obesity and physical activity can affect muscle mass and platelet function via their regulatory effect on inflammatory state in the elderly population [45–48]. Finally, in model 4, we adjusted the association between sarcopenia and PLT by Hgb, triglyceride, creatinine. Hgb level decrease in age-related inflammation, which might predispose the elderly population to sarcopenia by decreasing tissue oxygenation [49–51]. Triglyceride level is associated with a decrease in muscle mass, and it also might affect other sarcopenia parameters via MetS [32, 52]. Creatinine level approximately represents whole-body muscle mass and shows the renal function and related conditions such as CKD, which previously has been shown to be associated with muscle mass decline and sarcopenia [31, 53, 54]. We summarized the association between variables in Fig. 4.

Previous findings revealed that the biological effect of aging might be caused by oxidative stress and mitochondrial dysfunction [55]. The aging process is associated with chronic low-grade inflammation, which is called Inflamm-aging [56]. It has been demonstrated that Inflamm-aging plays a prominent role in the pathogenesis number of age-related chronic diseases such as atherosclerosis, insulin resistance, sarcopenia, frailty, and disability [57]. For instance, obesity and body fat mass, which have been explained to be associated with sarcopenia, have been shown to be positively correlated with PLT activity [18].

In our study, severe sarcopenia participants had a higher amount of PLT and PWR than other groups. After adjusting the model, the effect of PLT on sarcopenia showed a meaningful effect; however, the pathway of this effect is not clearly understood.

This association might be explained by endothelial dysfunction. Endothelial dysfunction is identified as an imbalance between vasodilatory and vasoconstrictive actions that cause a reduction in vasodilation activity of vessels. The integrity of endothelium, different receptors, and flow-mediated stimuli can affect the production and release of endothelium-derived relaxing factors (EDRF) such as nitric oxide [58]. EDRF causes vasodilation by activated cellular cascade such as soluble guanylate cyclase and subsequently raises cyclic guanylate in vascular smooth muscle. EDRF can play an anti-inflammatory role by inhibiting PLT aggregation and adhesion. Moreover, PLTs are initiators of vascular inflammation and remodeling and might cause oxygen and nutrients supply disturbance in muscle cells by interfering in microcirculation and microvascular endothelium [59, 60]. Another possible mechanism is that PLTs might accelerate bone marrow hematopoietic stem cell proliferation and affect the differentiation of human CD34-positive cells into foam cells, which has been shown to play a key role in the pathophysiology of atherosclerotic and small vessels diseases such as cerebral microbleeds (CMBs) [60, 61]. CMBs have been shown to be associated with cognitive impairment, physical frailty, and low handgrip strength, independent of other confounding factors. This hypothesis might be confirmed by a higher prevalence of cognitive impairment in severe sarcopenic participants.

White blood cell count is a strong indicator of low-grade inflammation and also might interfere with muscle metabolism by producing pro-inflammatory cytokines like IL-6 [20, 25]. However, our study did not find any significant association between WBC count and sarcopenia; even PLT and PWR have been shown to have a significant association.

Effect of PLTs and WBC count can be simultaneously assessed by PLT to-white blood cell ratio (PWR) [62]. The interaction between PLTs and WBC has been introduced in the pathogenesis of several diseases (e.g., cerebrovascular infarction). PLTs have been shown to affect other blood cells by releasing chemokines and membrane ligands and also play as a bridge in white blood cells-PLT aggregates (LPAs) in the periphery [63]. Therefore, PWR can represent the degree of inflammation, and a significant association of PWR may show the prominent effect of PLT than WBC on sarcopenia.

PLT count has been shown to decrease with aging in different older adults’ population. It has been demonstrated that despite the stable number of PLTs in middle-aged people, the PLT count declines by approximately 10% after age 60, which is more prominent in men [64]. Previous studies have shown that women have almost 15% higher PLT count than men, showing gender differences in PLT count in older adults [64, 65]. However, our study showed that PLT count might decrease slightly after 60 in women. Although the PLT count in men is lower than women, we see the gradually escalating change in PLT count after age 90 in men. However, only less than 0.6% of our population is older than 90 years; therefore, we cannot interpret this increasing trend accurately. PLT count decreased in the older adults might be explained by the fact that hematopoietic stem cell reserve reduces with aging [66]. Although the drop in PLT count is observed, PLT activity seems to increase with aging. It shows that the level of cytokines released by activated PLTs such as PLT factor 4 (PF4), which affects muscle mass, increases in the older adults population [59, 67]. The effect of age-related inflammation might explain this increase. In addition, it has been revealed that platelet response to inflammatory cytokines increases by aging, leading to platelet hyperactivity in older adults [68].

Some previous studies have classified PLT into quantile or tertial, for instance, in the National Health and Nutrition Examination Survey (NHANES) III, it was shown that elevations in serum PLR values were significantly associated with sarcopenia status and negatively associated with skeletal muscle index. After additionally adjusting for other covariates, the significant negative correlation remained. Participants with the highest quartile serum PLR value had a greater risk of sarcopenia than those with the lowest quartile [26]. Also, the West China Health and Aging Trend (WGHAT) study showed that participants in the highest tertial NLR, PLR value group had higher odds for sarcopenia than those in the lowest tertial value group [20]. In addition, Lee’s study, which was done in Korean National Health and Nutrition Survey, showed that higher PLT and WBC tertial are associated with sarcopenia [18]. All of these studies were done on the population-based study, and their *P*-value for trend showed significant results for the highest group. For this reason, the results of our study can indicate that the results of our study are evidence-based.

After stratifying participants based on gender in this study, none of the sarcopenia parameters had a statistically significant association with PLT in men. These results were consistent with previous studies emphasizing gender-related differences in sarcopenia and sarcopenia parameters [69, 70]. However, the differences might be related to the age-related decline in sex hormones and the other physiological pathway, especially in menopausal women, who have a higher decline rate than men [71]. It has been confirmed that the decline in estrogen levels in postmenopausal women is associated with a decrease in muscle mass. This association could be explained by the regulatory effects of estrogen on pro-inflammatory cytokines and the direct protective effect of estrogen on muscles, which decreases in menopausal women. Gender-related variations in the distribution of fat mass might also be attributed to these differences. Furthermore, in developing countries such as Iran, women have been shown to be frailer, making them more susceptible to sarcopenia than men [72]. Higher prevalence of frailty in women might be due to the fact that a significant number of the women’s population in lower-middle-income (LMICs) and developing countries, have a lower socioeconomic status than men, which might affect the quality of their nutrition intake, chance to participate in regular exercise, and access to healthcare services.

Moreover, after adjusting medications (e.g., anti-inflammatory, anti-PLT, HTN, anti-hyperlipidemic, and anti-diabetic medication) in women, results demonstrated that the association between PLT, ASM, and handgrip strength decreased, so that it was no longer statistically significant in the handgrip strength. These medications might prevent sarcopenia and might directly reverse this process. According to Landi et al., individuals who used non-steroidal anti-inflammatory drugs (NSAID) had almost 80% lower risk of sarcopenia compared with non-NSAID users, even after considering potential confounders [40]. Furthermore, anti-diabetic agents such as metformin play a protective role against sarcopenia through increased insulin sensitivity and glucose hemostasis. Aghili et al.’s study showed that those who received metformin had a lower risk for sarcopenia, which was notably true in women [73, 74].

Compared to previous studies, we used both upper and lower muscle strengths according to the revised edition of the European Working Group on Sarcopenia in Older People (EWGSOP2), which might be a better indicator for the decline in muscle strength in the elderly population because it simultaneously assesses both upper and lower limbs. In this study, when sarcopenia was defined to only mean handgrip, the prevalence of sarcopenia was 27.17%, while considering both upper and lower muscle strengths, the prevalence was 35.89%. According to supplemantory 1, which recaps previous related studies, the prevalence of sarcopenia among the elderly was 9.9 to 45.8%. When sarcopenia was defined based on other criteria in this study, it had almost the same prevalence as previous studies. The prevalence of sarcopenia according to different criteria was 27.87% for the foundation for the National Institutes of Health (FNIH); 30.20% for Asian Working Group for Sarcopenia (AWGS); 34.20% for International Working Group on Sarcopenia (IWGS); 45.36% for AWGS 2019.

This study was conducted using reliable data and a fully validated protocol with a large number of participants representing the Iranian older adults’ population. In this study, sarcopenia was defined by the revised edition of the European Working Group on Sarcopenia in Older People (EWGSOP2).

One of the most important strengths of this study is that most previous studies (supplementary 1) have focused on secondary sarcopenia, which is considered when factors other than aging are evident, especially systemic diseases such as malignancy or organ failure. However, in this study, we investigated sarcopenia as the primary outcome, which means no other specific cause is evident. This population-based study was done on a large sample of the elderly population, consisting of both genders. We did not exclude participants with underlying chronic diseases or those who had platelet counts < 150 × 103 and > 450 × 103 or WBC < 3000 or > 10,000 cells/μl or those diagnosed with cognitive impairment, also we believe that we encountered less selection bias which previous studies may have encountered. Another strength of this study compared to the previous studies is that in addition to muscle mass, we assessed both upper and lower muscle strengths, which might better indicate elderly condition and sarcopenia than using only the upper limb (handgrip). Also, in this study, determining a specified cut-off point helped to evaluate the Iranian elderly precisely, and our community, as one of the districts with the highest sarcopenia prevalence in the world, might be one of the best places for more investigating the sarcopenia etiology, and we may find the prognostic factors for future emergency implementation. Moreover, in this study, we used multivariable analysis procedures, including anti-inflammatory, anti-platelet, HTN, and DM medication, which helps to understand that these drugs might help prevent sarcopenia. We believe that using these can help clarify the association between sarcopenia and inflammatory markers by considering a number of covariables, which were not used entirely in the previous studies, and help enhance a better understanding of this association.

This study had a number of potential limitations, one of which was the cross-sectional nature of the study since musculoskeletal outcomes were measured only in the second stage of the BEH program, and thus, the cause-effect relationships between PLT, PWR, and sarcopenia could not be recognized. In addition, other inflammatory cytokines and markers (e.g., TNF-a, interleukin- 6, C-reactive-protein), mean PLT volume (MPV), and nutritional status were not measured in this study.

## Conclusion

Since most inflammatory factors have an inflammatory basis in old age, such as tumor necrosis factor α (TNFα), interleukin 6 (IL-6) and C-reactive protein (CRP), and activation of the inflammatory process play a role in the occurrence of these conditions, several factors may be used as indicators to show the inflammatory milieu. This study showed that PLT and PWR were associated with sarcopenia after considering confounding factors, muscle mass, and muscle strength, whereas WBC was not significantly connected with sarcopenia. Moreover, based on the results, women showed a significant association with PLT levels and sarcopenia with their components of it.

We investigate the association between age-related sarcopenia in an elderly population-based study. While most previous studies have focused on secondary sarcopenia, the present population-based study aimed to find sarcopenia in the population and evaluate the age-related sarcopenia disorder; therefore, for the first time, we found that PWR can be a prognostic marker for sarcopenia as previous studies have shown this inflammation process. We concluded that the elderly might produce an inflammation milieu, and PWR may be one of the most important inflammation markers for diagnostic age-related sarcopenia.

The association between PWR and sarcopenia is known to be independent of other predictors. Therefore, evaluation of PWR values may help in the early detection of elderly patients with sarcopenia. A notable feature of this ratio is that an easy, common, and available measurement may be an early, convenient, and important identification tool for sarcopenia in the elderly. Nonetheless, further longitudinal studies with different inflammatory cytokines are needed to confirm the connection between the inflammatory markers and sarcopenia and whether anti-inflammatory medication can prevent sarcopenia from happening.

## Supplementary Information

**Additional file 1: Supplementary 1**. Characteristics of previous studies worked on sarcopenia and inflammatory marker.

## Data Availability

The datasets used during the current study are available from the corresponding author, AO (a.ostovar@bpums.ac.ir) or IN (inabipour@gmail.com) upon reasonable request.

## Abbreviations

BEH: Bushehr elderly health
SBP: Systolic blood pressure
DBP: Diastolic blood pressure
BMI: Body mass index
WHR: Waist to hip ratio
IADL: Instrumental activities of daily living
ASM: Appendicular skeletal muscle mass
SMI: Skeletal muscle mass index
SPPB: Short Physical Performance Battery
HTN: Hypertension
HDL: High-density lipoproteins
LDL: Low-density lipoproteins
TG: Triglycerides
Hgb: Hemoglobin
RBC: Red blood cells
PLT: Platelet
WBC: White blood cells
PWR: PLT to WBC ratio
BUN: Blood urea nitrogen
ALK-P: Alkaline phosphatase
HbA1c: Hemoglobin A1c
EDRF: Endothelium-derived relaxing factor
