# Supplementary material for: Association between platelet, white blood cell count, platelet to white blood cell ratio and sarcopenia in community-dwelling older adults: focus on Bushehr Elderly Health (BEH) program
Source: BMC Geriatr. 2022 Apr 8;22:300. doi: 10.1186/s12877-022-02954-3 (PMC8991783; doi:10.1186/s12877-022-02954-3)
Supplement: Supplementary file 1 — Additional file 1: Supplementary 1. Characteristics of previous studies worked on sarcopenia and inflammatory marker. [file 12877_2022_2954_MOESM1_ESM.docx]

| supplementary 1: Characteristics of previous studies worked on sarcopenia and inflammatory marker | | | | | | | |  |  |  |
| --- | --- | --- | --- | --- | --- | --- | --- | --- | --- | --- |
| Study name | method | | | | Result | | | | Discussion | |
|  | Number of participants | Method for measuring muscle mass | Sarcopenia definition | Inflammatory Parameter | Sarcopenia prevalence | association | model | | Detail | Strength &  Limitation |
| [17] | - 2152 **post-menopausal Korean women** - Excluded women with chronic kidney disease, chronic liver disease | - Muscle mass was measured by DXA | Sarcopenia was defined as an ASM divided by Wt (ASM/Wt) that was greater than1 SD below the mean of a sample of 1415 women from the same2010 and 2011 KNHANES database. | WBC | The overall prevalence of **sarcopenia was 35.1%** | Highest quartile for sarcopenia (over 6610) was associated in crude analysis, and remain significant after adjusting. | - age, waist circumference, cigarette smoking, alcohol intake, regular exercise, blood pressure, fasting plasma glucose, triglyceride, and HDL-cholesterol. | | - In this study, association was assessed only in post-menopausal women | leukocyte counts over 10,000 were excluded from the analysis  CRP was not measured, cross-sectional design  **Muscle strength was not used in sarcopenia definition.** |
| [27] | 3,671 | BIA | - Muscle strength was not used - Was defined based on   sex-specific  cutoffs proposed by Janssen et al. | **PLR** | - class I sarcopenia and class II sarcopenia were **61.8%** and 19.7% in male patients, and   28.8% and 14.8% in female patients | - PLR negatively associated with the SMI - higher PLR were at higher risk of sarcopenia   in quartiles analysis, higher PLR levels exhibited a greater risk of sarcopenia | **Model 1** = age,  gender, race. **Model 2** = Model 1 + (stroke, heart disease, metabolic syndrome, current smoker). **Model**  **3** = Model 2 + (white blood cell count, haemoglobin, C-reactive protein, fibrinogen, uric acid, bilirubin,  creatinine, albumin, vitamin D). **Model 4** = Model 3 + (gait speed) | |  | - cross-sectional nature of the study - **Muscle strength was not used in sarcopenia definition,** - different automated hematological analysers can also yield varying neutrophil, lymphocyte, and   platelet counts |
| [18] | 639 patients | DXA |  | - ESR - Ferritin   CRP | 15.81% | Higher WBC, ESR was associated with sarcopenia and obesity sarcopenia, while CRP was only significantly associated with sarcopenia. | The model was  adjusted by the covariates age, sex, ADL, MNA, and MMSE | | - Exclude criteria:   MMSE < 18 points), heart and kidney dysfunction, |  |
| [23] | 670 of gastric cancer patients | A cross-sectional CT image of the third lumbar vertebra  (L3) was selected for valuing muscle mass | - sarcopenia was diagnosed based on   1) muscle mass (L3 SMI ≤40.8 cm2/m2  for men and 34.9 cm2/m2 for women); 2) muscle strength (hand grip strength <26 kg for men and <18 kg for women);  and 3) muscle performance (6 m usual gait speed <0.8 m/s). | - NLR - PLR - Large PLT to lymph - Lymph | 15.5% | in patients  with sarcopenia, NLR, PLR, and LPLR were significantly higher in patients with gastric  cancer  in Univariate analysis,  NLR, and PLR  showed significant differences according to  sarcopenia status  NLR, and PLR were independent predictors for sarcopenia in patient with gastric cancer | Univariate analysis and multivariate analysis (Fourteen variables from univariate analysis (P<0.05) were  chosen for multivariate) | | - exclusion criteria were occurrence   of another malignancy during the 3 years prior to surgery;  an emergency operation; preoperative chemotherapy or  radiotherapy; severe bleeding or immune system disease;  severe inflammation, such as secondary peritonitis. | - large sample size and   prospective data collection.   - unable to disentangle the network   of two-way relationships among inflammation, sarcopenia,  and cancer.  sarcopenic obesity  may have been ignored  due to defined 18 as the cutoff point  of BMI |
| [24] | 419 | BIA | - use EWGSOP algorithm and Turkish population-based cut-off points for   diagnosis of sarcopenia | **NLR** | 25% | One unit  increase in NLR resulted in 1.31 fold risk increase for sarcopenia  There was a statistically significant  positive correlation between NLR and CRP levels in sarcopenic group  There was a statistically significant association between NLR and sarcopenia in univariable analysis | **univariate binary logistic regression model.** | |  | - cross-sectional nature of the study - **the relationship between elevated NLR**   **and co-morbid conditions may have confounded by some unmeasured**  **covarities.** |
| [22] | - 2810 postmenopausal   Women | - DXA | Sarcopenia was defined by ASM/BMI <0.512 | - PLT | - The prevalence of sarcopenic obesity was 20.0% | - 4^th^ quartile, PLT was associated with sarcopenic obesity, **in crud analysis** and adjusted analysis | - Fully-adjusted model: age, systolic blood pressure, HOMA-IR, total cholesterol, triglyceride, total calorie intake, regular exercise, current smoking, and education   levels. | | - quartiles range: Q, 150–222; Q2, 223–257; Q3, 258–294; and Q4, 295–450 (103/μl). - In this study, association was assessed only in post-menopausal women - **platelet counts <150×10^3^ and >450×10^3^ were** excluded | cross-sectional design, **Muscle strength was not used in sarcopenia definition,** CRP was not measured, **medication affecting was not considered.** |
| [25] | 69 patients | CT of third lumbar vertebra(L3) | - Cutoff   L3MI values were defined as 52.4 cm2/m2 for males and  38.5 cm2/m2 for females, | NLR | - 30% | In univariate analysis, sarcopenia, performance status, and  serum cytokeratin-19 fragment levels were predictors of poor prognosis;  In multivariate analysis, performance status  and sarcopenia were independent predictors of poor prognosis. | Univariate and  multivariate | | - The inclusion criteria were who underwent definitive pulmonary resection   with mediastinal lymph node dissection for primary  lung cancer | it was a retrospective  study, small number of  patients and may not be applicable   - to a general population |
| [19] | - 10,092 participants | - Muscle mass was measured by DXA | Sarcopenia was defined ASM/BMI ratio (<0.789 for men and <0.512 for women) | PLT  WBC | Women (T1=6.8%; T2=7.0%; T3=10.1%)  Men (T1=6.8%; T2=7.8%; T3=10.9%) | 3^rd^ tertiles PLT and WBC was associated with sarcopenia in men and women, after adjusting | age, smoking, alcohol intake, hypertension, dyslipidemia, and **number of chronic diseases** | | - subjects with   leukocyte counts <3000 or >10000 cells/μl and subjects with platelet counts <150×10^3^ and >450×10^3^ were excluded | - cross-sectional design, **Muscle strength was not used in sarcopenia definition,** CRP was not measured |
| [20] | - **442** participants | - Muscle mass was measured by BIA | - Sarcopenia definition based on the EWGSOP2 definition cut-off points, | - ESR - Albumin - WBC |  | - In crude analysis, higher WBC was significantly associated with lower gait speed   and handgrip strength, while, after adjustment the associations were no longer significant. |  | | - In this study, association between WBC and **measures of sarcopenia was assessed** - No exclusion criteria were applied | - considering anti-inflammatory medication in analysis. - CRP was not measured, cross-sectional design |
| [26] | - 384 participants | - BIA | Sarcopenia was defined based on AWGS (2019), EWGSOP2, IWGS | - PLR - NLR - LMR | The overall prevalence of sarcopenia was varied from **9.9% (EWGSOP2)** to **45.8% (AWGS 2019).** | No significant association between PLR, NLR,  LMR in AWGS 2019, EWGSOP2 | In this study, analysis model was **Model 1:** adjusted for age and gender. **Model 2:** model 1+ coronary heart disease, and cognitive impairment. **Model 3:** model2 + albumin, HDL-C, and BMI | | In this study, inflammatory index was note used independently | cross-sectional design, sample size, |
| [21] | 4224 participants | BIA | Sarcopenia was defined based on AWGS | - WBC - NEUT - LY - PLT - NLR - PLR | - The overall prevalence of sarcopenia was 19.3 % | - Sarcopenia was not associated with PLT and WBC in adjusted analysis, while in crude analysis was associated   WBC   - In tertiles analysis, PLT was only associated with sarcopenia in 3^rd^ tertiles in fully-adjusted model - NLR, and PLR were significantly associated with sarcopenia | Model 1 was adjusted for age, sex and ethnicity. Model 2 included Model 1 + smoking, alcohol drinking, number of chronic diseases, | | - Participants with chronic liver or   kidney diseases were excluded | cross-sectional design, CRP was not measured. |
| [28] | 64 patients | CT | - sarcopenic group (PMI   <5.5 cm2/m2 in men or <4.0 cm2/m2 in women) | - PLR | - 25.0% | PLR value >= 292.5 was shown to be a diagnostic marker for sarcopenia based  on the ROC curve (sensitivity 31.3%, specificity 91.7%).  Higher  PLR was significantly associated with elevated cases  of sarcopenia |  | | - The inclusion criteria were patients   who underwent infrainguinal surgical revascularization  and (2) patients in whom preoperative  CTA and blood analysis including platelet and  lymphocyte counts were performed | study design is retrospective **Muscle strength was not used in sarcopenia definition**  sensitivity 31.3%,for PLR cut-off point |
| Note: ESR: erythrocyte sedimentation rate; WBC: White blood cells; CRP: C-reactive protein; DXA: dual-energy x-ray absorptiometry; BIA: Bio-electrical Impedance Analysis or Bioimpedance Analysis; EWGSOP: European Working Group on Sarcopenia in Older People; AWGS: Asian Working Group for Sarcopenia; IWGS: International Working Group on Sarcopenia; PLT Platelets; NEUT: neutrophils; LY: lymphocytes; LMR: lymphocyte-to-monocyte ratio; PLR: platelet-to-lymphocyte ratio; NLR: neutrophil-to-lymphocyte ratio; LPLR: large platelet to lymphocyte ratio; ASM: Low appendicular skeletal muscle mass. | | | | | | | | | | |
